# Influence of arch form on the accuracy of digital workflow in all-on-four implant rehabilitation cases (an in vitro study)

**DOI:** 10.1186/s12903-026-09780-8

**Published:** 2026-09-15

**Authors:** Mohamed S. Torky, Rewaa G. AboElHassan, Amir S. Azer

**Affiliations:** Department of Conservative Dentistry, Fixed Prosthodontics , Faculty of Dentistry, Alexandria University, P. O. Box: 21527, Champollion St., Azarita, Alexandria, Egypt

**Keywords:** Arch form, Scanning strategies, Intraoral scanning, Accuracy, Digital impressions

## Abstract

**Background:**

Variations in arch form may influence the accuracy of digital impressions for implant-retained fixed dental prostheses. Differences in arch width, curvature, and span length can affect scanning continuity and data stitching, potentially impacting trueness and precision during full-arch acquisition. However, the extent to which arch form and scanning strategy interact to influence digital impression accuracy remains unclear.

**Purpose:**

The purpose of this in vitro study was to evaluate the effect of arch form on the accuracy of the digital work flow of implant-retained fixed dental prostheses.

**Methods:**

Three maxillary edentulous full-arch models with different forms—ovoid (O), tapered (T), and square (S)—were 3D printed. Four implants were placed in each model and loaded with scan bodies. Reference scans were obtained using a high-precision laboratory scanner. Each model was scanned with a Medit i700 intraoral scanner (IOS) using three scanning strategies Linear (L), S-curve (S), and Wave (W). Each strategy was performed 10 times per model, generating 90 STL files. Trueness and precision were evaluated using Geomagic Control X software and statistically analyzed (α = 0.05).

**Results:**

Arch form significantly influenced trueness (*p* < 0.001), with the tapered arch demonstrating the highest trueness, followed by square and ovoid forms. Precision was significantly affected by arch form (*p* < 0.001) and scanning strategy (*p* = 0.006). The wave strategy showed significantly greater precision than the other strategies. No significant interaction was found between arch form and scanning strategy (*p* = 0.769).

**Conclusion:**

Within the limitations of this in vitro study, Arch morphology and scanning strategy significantly impact both the trueness and precision of full-arch implant scans. While tapered arches demonstrated statistically highest trueness and precision and the wave scanning strategy maximized precision, all tested combinations remained safely within the clinically acceptable threshold for passive prosthetic fit.

## Highlights

Selection of the scanning strategy in case of all on four implant supported restorations should be accurately considered especially with different shapes of arch form, to enhance the precision of digital impression.

Tapered arch forms yielded significantly more accurate digital scans, irrespective of the scanning strategy used.

Tapered arches are associated with improved precision in digital impressions.

Wave scanning strategy are associated with improved precision in digital impressions among the different scanning strategies.

## Introduction

Dental arch shapes are generally classified as tapered (or narrow), ovoid, or square. A demographic study found that 41% had ovoid arches, 39% had square arches, and 20% had tapered arches [1]. The dimensions and shape of the dental arch could influence the precision of scans taken by intraoral scanners (IOSs) [2]. In previous studies on 3Shape Trios intraoral scanner (IOS) scans, it was discovered that the width of the arch significantly affects the accuracy, but not the authenticity of the scans. Scans of broader arches were less accurate [3]. 

Recent advancements in IOSs and enhancements in scanner precision have facilitated the creation of implant-supported prostheses using a direct digital process [4]. This approach has been clinically validated for its accuracy and has contributed to reduced discomfort for patients [5]. Additionally, a comparison of intraoral and extraoral scans revealed that the most significant differences were found in the posterior ridge areas, as opposed to the front region [6]. 

The use of IOSs for capturing the 3-dimensional shape of objects has seen a growing interest, since the introduction of digital workflow in dentistry. The precision of IOSs can be influenced by various factors such as the scanning approach, ambient lighting, the skill level of the operator, the design of the preparation, distance, hardware, and the version of the software program [7]. Additionally, the accuracy can be compromised by factors like the need for rescanning and stitching processes, the speed of scanning, the technology used for scanning, the traits of the surface being scanned, contamination from blood or saliva, the size of the dental arch, and the presence of misaligned teeth [8]. 

The scanning strategy involves the meticulous movement of the IOS head over the object being scanned, ensuring the object remains central in the viewfinder [9]. Typically, the process begins with the scanning of the arch designated for restoration, followed by the opposing arch, and concluding with the capture of the occlusal registration [10]. These findings indicate the importance of selecting an appropriate scanning strategy to ensure optimal precision in the digital workflow.

One of the recent IOS that was released is Medit i700 IOS. The Medit i700 IOS is recognized for its high performance, fast scanning speed, and user-friendly features, when compared to other scanners in the market, thus used to scan and export scans, which can then be used with compatible computer-aided design (CAD) software [11]. This flexibility allows dental professionals to choose their preferred workflow and software tools [11]. 

While scanning strategies have been researched, their interaction with specific arch forms in the context of the All-on-Four protocol remains under-reported. Therefore, ongoing research continues to explore different clinical conditions to determine the impact of various elements on scanning accuracy, including the arch form scanned and scanning strategy. The aim of this in vitro study was to examine the effect of three different arch forms (ovoid-tapered-square) for implant-retained fixed dental prostheses using different scanning strategies on the accuracy of the digital workflow. The null hypothesis was that there was no difference in the accuracy of all-on-four digital impressions in the three arch forms while using different strategies.

## Materials and methods

Three maxillary edentulous models representing actual human models with different arch forms ovoid (O), tapered (T), and square (S) were obtained from patients presented to the Department of Conservative Dentistry, Faculty of Dentistry, Alexandria University. The models were initially scanned using a desktop laboratory scanner (T710; Medit Corp., Seoul, South Korea), and subsequently fabricated using a 3D printer (Form 3+; Formlabs) according to the manufacturer’s guide.

The sample size was estimated using G*Power software (version 3.1.9.7), assuming a 95% confidence level (alpha = 0.05) and 80% statistical power (beta = 0.80). Based on the findings of Pattamavilai et al. [12], a medium-to-large effect size (f = 0.375) was utilized. For a Two-Way Analysis of Variance (ANOVA) (fixed effects, special, main effects, and interactions) with 9 total groups and 4 degrees of freedom for the interaction, the minimum required sample size was determined to be 90 (10 samples per group). sample was calculated by Gpower [13]. Although the controlled in vitro environment eliminates clinical confounding factors such as moisture and patient movement, a sample size calculation (*n* = 10 per group) was executed. This was necessary to ensure adequate statistical power to detect true micrometer-level geometric discrepancies between the scanning paths and arch morphologies, minimizing the risk of Type II statistical errors.

The scanned data were imported into dental CAD software (exoplan; exocad GmbH, Darmstadt, Germany) and used to design 3D-printed surgical guides for implant placement. To eliminate implant positioning as a confounding variable, a standardized implant distribution was planned in “exoplan” software and digitally synchronized across the three arch form models. Spatial uniformity was confirmed across arch forms by identical bilateral and cross-arch dimensions: the linear cross-arch distance between the two terminal posterior implants was exactly 43.366 mm, the distance between the two anterior implants was exactly 27.991 mm, and the anteroposterior distance between the adjacent anterior and posterior implants was exactly 18.400 mm. This rigorous dimensional standardization ensures that any observed variations in scanning accuracy are strictly attributable to the geometric transition curvatures of the arch forms themselves. This master plan was executed using printed fully guided surgical templates, ensuring identical inter-implant distances and angulations regardless of the arch morphology. Sleeved surgical guides were utilized to standardize the physical osteotomy and implant placement within the three resin casts, employing the NeoBiotech guided surgery kit (NeoBiotech Co., Ltd., Seoul, South Korea). This protocol enabled accurate control of implant site position, angulation, diameter, and depth [14]. 

**Fig. 1:**
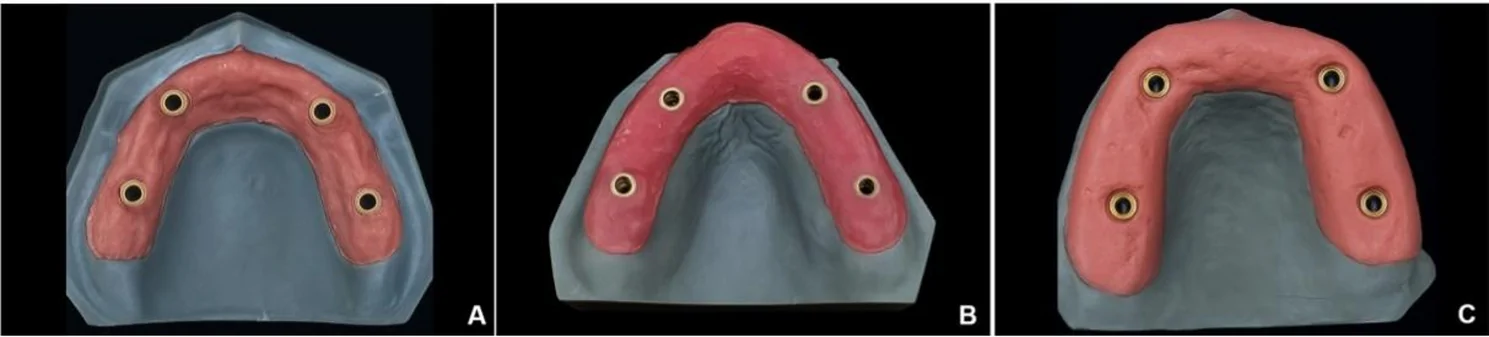
Three 3d printed maxillary edentulous arch forms with all on four implants. **A**, ovoid. **B**, Tapered. **C**, Square

In each model, four bone-level implants (Ø4.5 × 10 mm, IS-II Active; NeoBiotech Co., Ltd.) were placed: two at the canine regions in a straight (0°) configuration and two at the first molar regions with a 15° distal angulation, replicating the All-on-4 treatment concept [15]. Implant shoulders were aligned with the simulated alveolar crest of the porous bone material, reflecting typical clinical positioning [16] (Fig. 1).

**Fig. 2:**
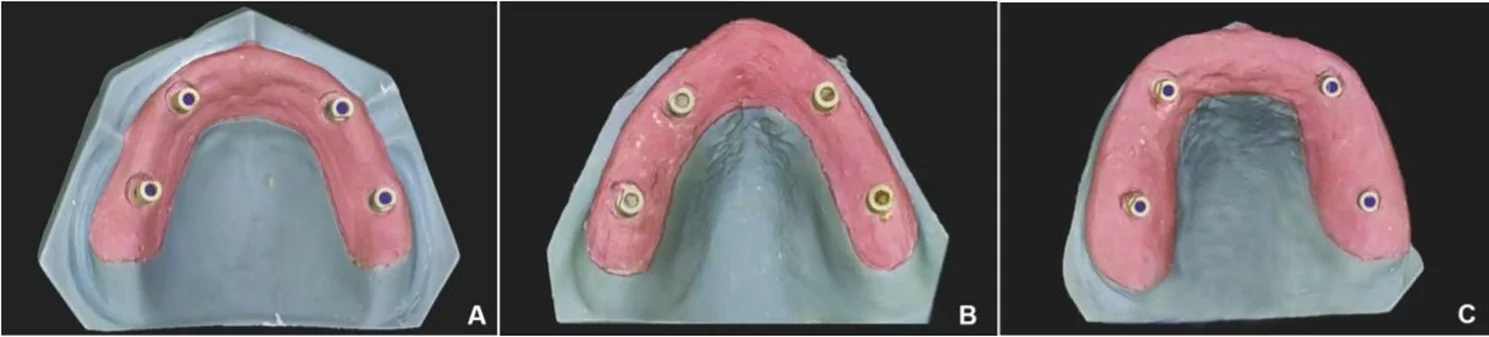
Scan bodies attached and torqued at implant level. **A**, ovoid. **B**, Tapered. **C**, Square

**Fig. 3:**
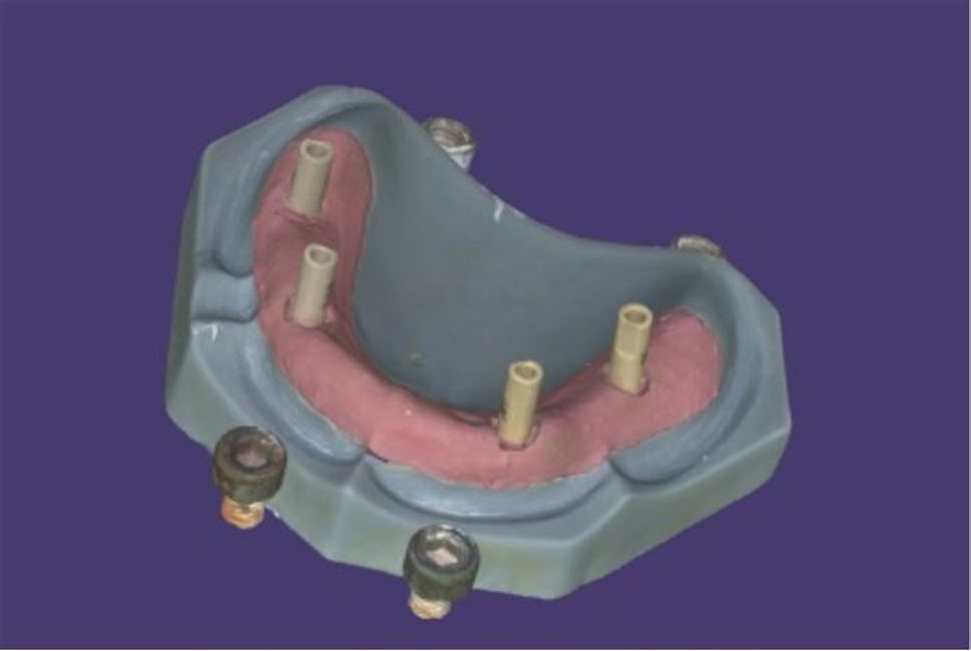
Reference scan by a high-precision laboratory scanner (T710; Medit Corp.)

After implant placement, implant-specific scan bodies (ScanBody for IS-II Active; NeoBiotech Co., Ltd.) were attached and torqued to 10 Ncm according to the manufacturer’s recommendations (Fig. 2). The models were rescanned using a high-precision laboratory scanner (T710; Medit Corp.) as a reference scan(C) under well-controlled conditions, then the digital files were exported in standard tessellation language (STL) format for subsequent analysis (Fig. 3).

The 3D-printed models were categorized into three main groups (O, T, and S), with each group further subdivided into three subgroups based on the scanning strategy employed: linear (L), S-figure (S), and wave (W). For each combination of scanning strategy and arch form, ten digital scans were acquired, following the methodologies described by Pattamavilai et al. [12] and Mai et al. [17] resulting in a total of 30 scan files per arch form. A well-trained operator with several years of experience scanned each group by Medit i700 IOS ((Medit Corp., Seoul, South Korea).) using three scanning strategies: (L-S-W). Each scan strategy was done 10 times for each model with a different arch form. All digital scans were scanned in the same room with the same light and humidity conditions. Before each new scan, the IOS was calibrated using the recommendation of the manufacturer’s guide. During the scanning procedure, Scans were only repeated in the event of a total software tracking loss or gross geometric artifacts that prevented the completion of the digital model. This protocol aligns with clinical standards where only complete, artifact-free datasets are acceptable for prosthetic fabrication [18, 19]. 

**Fig. 4:**
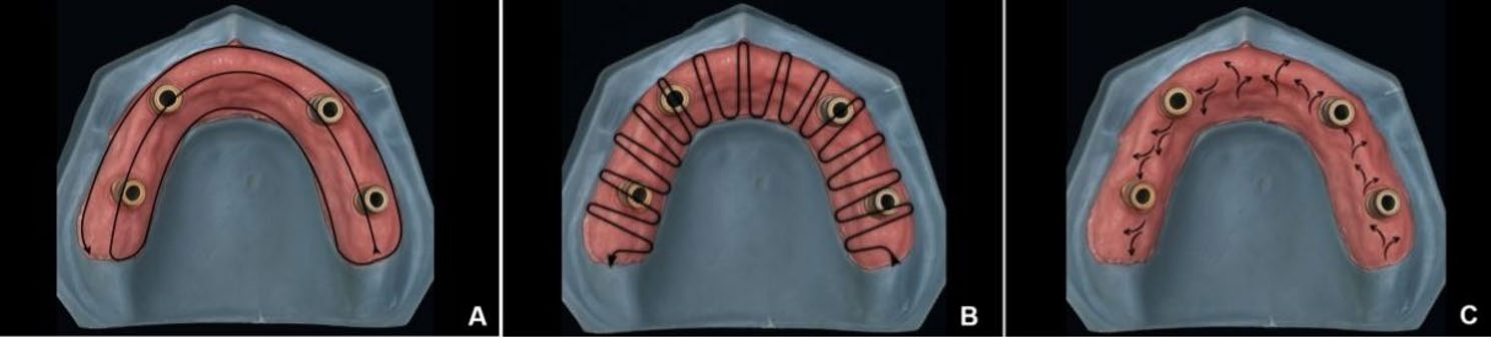
Three different scanning strategies. **A**, linear strategy. **B**, S. figure strategy. **C**, Wave strategy

The (L) intraoral scanning protocol was initiated at the distal aspect of the most posterior implant scan body and proceeded in a continuous, linear path across the occlusal/incisal surfaces of the arch from one posterior quadrant to the other. Following this, the scanner was angulated palatally to capture the palatal surfaces of the scan bodies and surrounding soft tissue, and then rolled buccally to record the buccal contours and vestibular anatomy, ensuring circumferential coverage of the arch [20] (Fig. 4A).

In the (S) scanning strategy, the scan began at the distal aspect of the most posterior implant scan body, similar to the linear approach. Following an initial occlusal pass, the scanner was manipulated in a dynamic, undulating motion that continuously transitioned between palatal and buccal aspects. This movement involved sweeping from the occlusal surface toward the palatal mucosa, then back across the ridge crest to the buccal vestibule, in a controlled S-like figure. This strategy was maintained throughout the arch to simultaneously capture palatal, occlusal, and buccal surfaces in one continuous motion. The inherent overlap produced by this sweeping path promoted robust image registration and minimized stitching errors. After the full arch was captured, the digital model was assessed for completeness, and touch-up scans were performed as necessary [21, 22] (Fig. 4B).

In the (W) scanning strategy, the scan began from a similar point as the two mentioned strategies. In this method, the scanner head was moved right and left in a rolling motion starting from the occlusal surface of the ridge to the buccal then again to the palatal surface and we repeated this motion to the full arch [21, 23] (Fig. 4C). Real-time image acquisition and stitching were conducted using the scanner’s software, with consistent frame overlap to ensure precise registration. The implant scan bodies functioned as critical markers, enabling accurate three-dimensional localization and angulation of the implants in the absence of natural teeth. Upon completion, the digital scan was evaluated for completeness and accuracy. All digital impressions were performed using a continuous, single-pass scan strategy. If an unrecoverable software tracking loss occurred, the scan was discarded and completely restarted. In cases where localized voids or surface artifacts occurred on non-critical anatomical zones, a targeted touch-up protocol was utilized: the specific defect was excised using the scanner’s native trimming tool, and selective scanning was performed to fill the void. This localized correction utilizes the surrounding point-cloud mesh as a rigid geometric reference frame, preventing the accumulation of global stitching errors or volumetric drift [24, 25]. 

A total of 90 (STL) files were obtained from the groups. The accuracy of digital impressions obtained using three different scanning strategies across three maxillary arch forms was assessed based on two key parameters trueness and precision by ISO 5725-1 guidelines. All analyses were conducted using Geomagic Control X software (3D Systems, Rock Hill, SC, USA) [26]. 

**Fig. 5:**
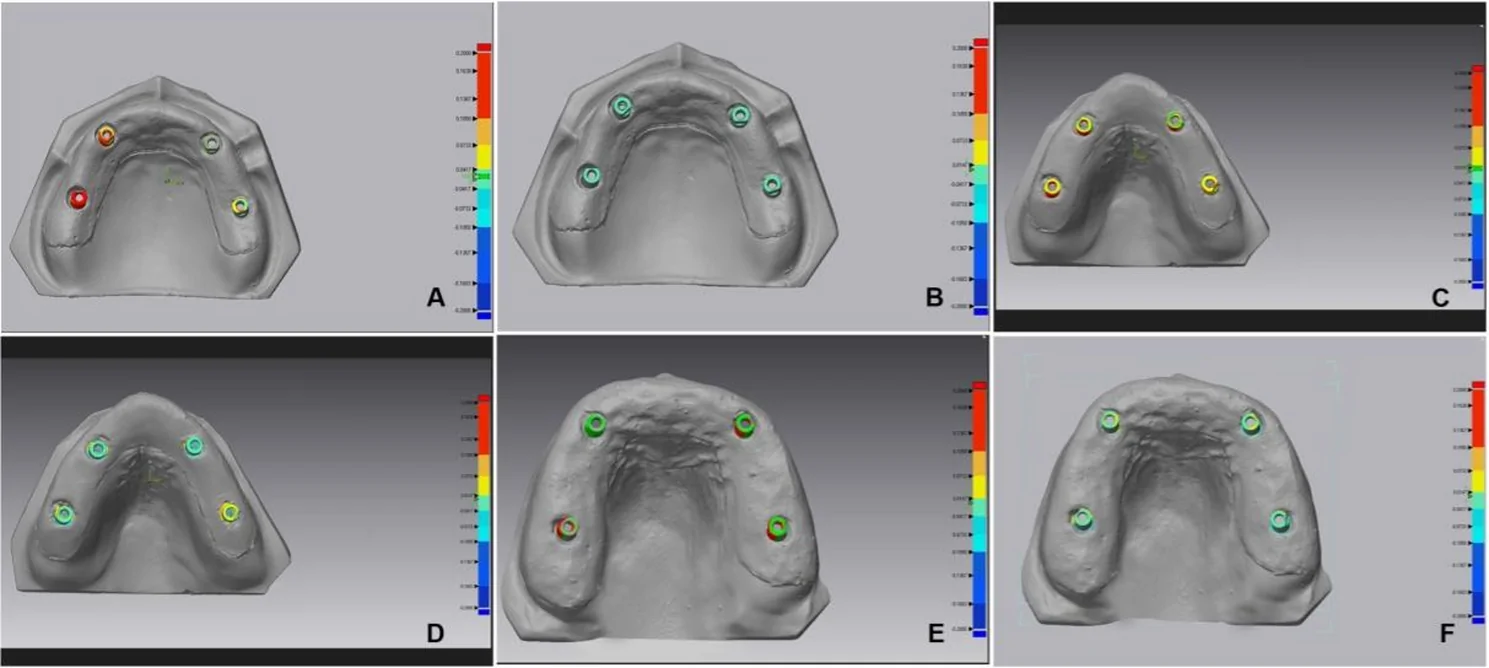
Heat Color maps representing 3D deviation of scan body position using geomagic control x software. **A**, **C**, **E**, Heat maps of three groups regarding trueness. **B**, **D**, **F**, Heat maps of three groups regarding precision

Trueness was evaluated by aligning each test scan with a pre-established reference scan through an initial alignment followed by a global best-fit registration. The deviation between the test scan and the reference scan was quantified using three-dimensional (3D) deviation analysis, and accuracy was expressed as the root mean square (RMS) error. Lower RMS values indicated higher agreement with the reference dataset, thereby reflecting greater trueness (Fig. 5A, C, E). Precision was assessed by performing pairwise comparisons among repeated scans within each scanning strategy group (*n* = 10 per group). Each pair was aligned using a best-fit algorithm, and RMS errors were calculated for all possible scan combinations within the same group. The mean RMS value across these comparisons represented the group’s precision, with lower values signifying greater consistency and repeatability (Fig. 5B, D, F). For both trueness and precision evaluations, 3D color deviation maps were generated to visually clarify the distribution of discrepancies across the full arch. A tolerance threshold of ± 50 μm was applied to classify deviations as within acceptable limits, positive, or negative [20, 22, 27, 28] (Fig. 5). RMS provides the quantitative value, 3D analysis provides the spatial context, and color maps offer the visual distribution of errors, these three elements provide a complete picture of accuracy.

To evaluate intra-examiner reliability across different scanning strategies and arch forms, 10 scan files were randomly selected and remeasured by the same examiner on two separate days. The intraclass correlation coefficients (ICCs) were 0.92 and 0.92, respectively, indicating excellent consistency.

### Statistical analysis

Normality of variables was checked using the Shapiro-Wilk test and Q-Q plots. Data were presented using mean and standard deviation (SD). To assess the effect of arch form and scanning strategy on trueness, precision. Pairwise comparisons were done with Bonferroni correction to adjust type I errors. All tests were two-tailed and the significance level was set at *p* < 0.05. Data was analyzed using IBM SPSS version 23 for Windows, Armonk, NY, USA.

## Results

The results demonstrated that among the arch forms, the tapered arch exhibited the highest trueness with the overall lowest mean, followed by the square arch and the ovoid arch (Table 1).

**Table 1:** Comparison of trueness (µm) values among the study groups

		Arch Forms			
		Ovoid (*n* = 30)	Square (*n* = 30)	Tapered (*n* = 30)	Overall
		Mean ± SD			
Scanning strategy	Linear	57 ± 2	55 ± 5	49 ± 5	54 ± 5
	Wave	54 ± 4	52 ± 5	47 ± 4	51 ± 5
	S-Figure	56 ± 5	52 ± 6	50 ± 5	52 ± 6
	Overall	55 ± 4	53 ± 5	49 ± 5	

A statistically significant effect of arch form on trueness values (F = 15.93, *p* < 0.001, Ƞp² = 0.282), while the scanning strategy (*p* = 0.069) and its interaction with arch form (*p* = 0.727) were not statistically significant (Table 2).

**Table 2:** Two Way ANOVA assessing the effect of arch forms and scanning strategy on trueness

Parameters	F test	*p* value	Ƞp^2^
Arch form	15.93	< 0.001^a^	0.282
Scanning strategy	2.77	0.069	0.064
Interaction	0.51	0.727	0.025

^a^Statistically significant difference at *p* value < 0.05, *Ƞp*^*2*^ Partial Eta Squared, Adjusted R Squared = 0.261

Pairwise comparisons revealed that trueness was significantly higher in the tapered arch compared to both the ovoid (mean difference = 7 μm, 95% CI: [4, 10], *p* < 0.001) and square arches (mean difference = 4 μm, 95% CI: [1, 7], *p* = 0.001), while the difference between ovoid and square arches was not statistically significant (*p* = 0.174) (Table 3).

**Table 3:** Pairwise comparisons regarding trueness (µm) values among different arch forms

Arch form	Compared to	Mean diff	95% CI	*p* value
Ovoid	Square	2	[-1, 5]	0.174
	Tapered	7	[4, 10]	< 0.001^a^
Square	Tapered	4	[1, 7]	0.001^a^

^a^Statistically significant difference at *p* value < 0.05

The study also revealed that among the arch forms, the tapered arch exhibited the highest precision, followed by the square and ovoid forms (Table 4).

**Table 4:** Comparison of precision (µm) values among the study groups

		Arch Forms			
		Ovoid (*n* = 30)	Square (*n* = 30)	Tapered (*n* = 30)	Overall
		Mean ± SD			
Scanning strategy	Linear	56 ± 2	54 ± 4	49 ± 5	53 ± 5
	Wave	52 ± 6	50 ± 5	42 ± 4	48 ± 7
	S-Figure	55 ± 6	51 ± 6	47 ± 5	51 ± 6
	Overall	54 ± 5	52 ± 5	46 ± 5	

Both arch form and scanning strategy significantly influenced precision values (*p* < 0.001 and *p* = 0.006, respectively), with no significant interaction effect between them (*p* = 0.769) (Table 5).

**Table 5:** Two Way ANOVA assessing the effect of arch forms and scanning strategy on precision

Parameters	F test	*p* value	Ƞp^2^
Arch form	22.75	< 0.001^a^	0.360
Scanning strategy	5.55	0.006^a^	0.120
Interaction	0.45	0.769	0.022

^a^Statistically significant difference at *p* value < 0.05, *Ƞp*^*2*^ Partial Eta Squared, Adjusted R Squared = 0.362

Pairwise comparisons confirmed that the tapered arch had significantly higher precision compared to both ovoid (*p* < 0.001) and square forms (*p* < 0.001), while no significant difference was observed between the ovoid and square arches (*p* = 0.138) (Table 6).

**Table 6:** Pairwise comparisons regarding precision (µm) values among different arch forms

	Groups	Compared to	Mean diff	95% CI	*p* value
Arch forms	Ovoid	Square	3	[-1, 6]	0.138
		Tapered	9	[5, 12]	< 0.001^a^
	Square	Tapered	6	[3, 9]	< 0.001^a^
Scanning strategy	Linear	Wave	4	[1, 7]	0.004^a^
		S-Figure	2	[-1, 5]	0.348
	Wave	S-Figure	-2	[-5, 1]	0.256

^a^Statistically significant difference at *p* value < 0.05

Regarding scanning strategy, the wave pattern yielded significantly better precision than the other strategies (Table 4).

## Discussion

This study was conducted to examine the effect of arch form on the accuracy of digital workflow using an intraoral scanner with different scanning strategies for implant-retained fixed dental prostheses. The preservation of distinct anatomical landmarks, such as the palatal rugae and surface morphology unique to each arch form, was a deliberate methodological choice. While these variations influence data stitching, they represent the biological reality of the three tested morphologies. Consequently, the observed differences in accuracy accurately reflect the interaction between the IOS scanning strategy and the specific geometric and anatomical constraints of each arch form. Our results indicated that tapered arch forms yield significantly more accurate digital scans, irrespective of the scanning strategy used. It also indicated that tapered arches and wave scanning strategies were associated with improved precision in digital impressions, so the null hypothesis was rejected. Crucially, a distinction must be maintained between mathematical statistical significance and clinical thresholds. While all tested scanning strategies fell within the accepted 50 μm threshold for clinical success, the statistically significant differences observed among the arch forms remain highly relevant. In a multi-stage digital workflow, the final prosthetic passivity depends on a cumulative error budget; minimizing initial intraoral scanning deviations by choosing an optimized strategy for specific arch form provides a vital safety margin, preventing downstream CAD/CAM milling and sintering tolerances from exceeding biological structural thresholds. Therefore, these small statistical variations are clinically meaningful as a protective buffer for the total workflow accuracy [29]. 

The results of this study contrast with previous reports suggesting that tapered arch forms are associated with lower scanning trueness and precision [30, 31]. Our findings indicate that the tapered arch exhibited the highest trueness and precision among the tested arch forms.

Although digital intraoral scanning has been shown to provide clinically acceptable accuracy for full-arch implant impressions when compared with conventional techniques, considerable variability remains across studies [32]. Anatomical features of the scanned arch, such as arch size and edentulous span, significantly affect precision, with larger areas posing greater challenges for IOS accuracy [33]. Additionally, scanning strategy and pattern have been shown to influence trueness and precision, indicating that both device-specific performance and operator-dependent protocols interact with arch morphology during digital impression acquisition [34]. One clinical comparative study even demonstrated a significant correlation between arch type and IOS accuracy, reinforcing the potential impact of arch form on scanning outcomes [35]. 

Several factors may account for this discrepancy like; there is a relation between arch form and scanner path/scan stitching accuracy. Intraoral scanners work by capturing multiple overlapping images that are stitched together to reconstruct a 3D model. The accuracy of this stitching depends on the shape complexity and scan path continuity. Tapered arches have a more continuous and convergent curvature toward the anterior region. This allows smoother and more predictable scan paths with less abrupt changes in curvature. Square arches, characterized by a wider and flatter anterior region, and ovoid arches, with a more rounded but broader shape, present a more complex geometry with varying curvature radius. This complexity can cause more distortion in the image stitching process due to the increased challenges in alignment and overlapping of scan data [36]. There is also an impact of arch form on implant scanning and all-on-four cases.

While the physical inter-implant distance remained strictly standardized across all groups, the tapered arch form presents a narrower posterior anatomical arch geometry and soft-tissue boundary. This configuration reduces the lateral volume of surrounding surface data that the intraoral scanner must stitch. Consequently, error propagation in full-arch scanning is multi-factorial, driven by a combined effect of favorable geometric curvature transitions around the canine eminence and reduced surrounding anatomical form. In contrast, square and ovoid customized arches with wider posterior anatomical arch geometry and soft-tissue boundary leading to more stitching errors and deviations [37]. 

Clinical studies comparing the accuracy of intraoral scanning in various dental arch forms have reported that arch form influences accuracy, with tapered and narrower arches tending to yield better scan results. The narrower tapered arch allows less cumulative distortion because the scanner sensor has less angular displacement needed and maintains optimal distance from tissue throughout the scanning procedure [38]. Advances in intraoral scanning technology, including improved calibration and software algorithms, may have enhanced the ability to capture the small detailed features of tapered arch forms more accurately. Additionally, the specific scanning protocols employed in this study, such as optimized scanning angles and sequences, could have been more conducive to capturing the geometry of tapered arches effectively. These findings clarify the importance of considering both arch morphology and scanning system characteristics when evaluating digital impression accuracy.

The present study also observed that the wave scanning pattern significantly enhanced precision compared to the other patterns, while trueness remained unaffected by the scanning strategy. These findings are consistent with recent literature highlighting the influence of scanning protocols on digital impression outcomes [39–41]. 

Recent studies reported that employing scanning processes using artificial landmarks and digital impressions under dry conditions can enhance the accuracy of digital intraoral scanners. Additionally, these studies found that an S-shaped pattern improved both trueness and precision. These results suggest that specific scanning strategies can positively impact the accuracy of digital impressions [39, 40]. Similarly, another pilot study investigated the effect of different scanning protocols on the accuracy of intraoral scanning. The study found that certain scanning protocols, such as wave-like movements, resulted in higher precision compared to others. However, trueness was not significantly affected by the scanning protocol [41]. The wave scanning strategy offers significant advantages for digital impression acquisition in complex cases, such as those involving “All-on-4” implant configurations [31]. This technique promotes enhanced data registration by utilizing a continuous, sweeping motion that generates extensive data overlap [42]. This comprehensive overlap is important for the scanner’s software to construct an accurate 3D model, particularly in long edentulous spans where anatomical landmarks are limited. Furthermore, the wave strategy actively reduces cumulative error by continuously re-registering and refining the acquired data through its overlapping sweeps and maintaining accuracy across the entire arch [43]. The varied angulation of this motion also contributes to improved soft tissue capture, which is vital for optimizing the emergence profile and fit of the definitive prosthesis. While operator skill influences its execution, the adaptable nature of the wave pattern allows for effective navigation of challenging anatomical features and areas with restricted line-of-sight. The underlying principle of the wave strategy is to ensure comprehensive, overlapping, and continuous data acquisition via its path. That’s why it is valuable for achieving high accuracy and precision in demanding clinical situations [44]. 

As an interaction between the arch form and scanning strategy, the present study highlighted the importance of selecting an appropriate scanning strategy to enhance the precision of digital impressions according to each arch form. Within the cumulative error budget framework, matching a specific scanning strategy to a tapered arch configuration may optimize the continuous optical tracking and stitching pathways of the software. By minimizing data fragmentation along that specific trajectory, this optimized pairing allows the initial digital impression stage to consume a smaller fraction of the total workflow error budget.

While trueness may not be significantly influenced by scanning strategy, precision can be markedly improved, leading to more reliable and consistent digital impressions. For the ovoid arch, the wave strategy achieved the lowest mean deviation values, indicating the highest trueness and precision compared to the linear and S-figure strategies. Similarly, in the square arch, the wave and S-figure strategies showed equal trueness, but the wave strategy had better precision, making it the preferred approach. In the tapered arch, the wave strategy again outperformed the others, showing the lowest mean values for both trueness and precision.

A primary limitation of this study that it was conducted under in vitro conditions, which intentionally isolates geometric variables but may not fully replicate the complexities of clinical practice. Patient-specific confounding variables—such as salivary flow, patient movement, and intraoral humidity—were absent, which may artificially optimize the tracking accuracy observed. Previous researches had indicated that IOSs often demonstrate higher accuracy in laboratory settings compared to in vivo applications [45–48]. The increased visibility available in a simulated setup may have led to an overestimation of IOS performance. Additionally, this study evaluated only a single IOS brand, as introducing multiple platforms into this specific protocol would have introduced significant confounding variables. Because modern digital impression systems rely on highly diverse optical capture technologies and distinctive proprietary stitching software, the exact interaction between arch geometry, scanning strategy, and the cumulative workflow error budget may vary across different commercial platforms.

Consequently, Further studies are recommended to explore the underlying mechanisms by using different IOSs and scanning strategies to determine the generalizability of these findings across different scanning technologies and patient populations.

## Conclusion

The results of the present study demonstrated that:

Within the limitations of this in vitro study, it can be concluded that the geometric configuration of the dental arch and the choice of scanning strategy are significant variables in the accuracy of digital impressions for ‘All-on-Four’ rehabilitations. While the tapered arch form showed the highest trueness and precision and the wave scanning strategy demonstrated the highest precision, all observed deviations remained within 50 μm threshold commonly cited for clinical success in implant prosthodontics. Therefore, the differences observed between arch forms should be interpreted as an optimization of the cumulative error budget and an enhancement of the surgical-prosthetic safety margin, rather than as a definitive indicator of clinical failure for any specific arch morphology.

## Data Availability

The datasets utilized and/or analysed during this study is available from the corresponding author upon reasonable request.

## Abbreviations

IOSs: Intraoral scanners
STL: Standard tessellation language
RMS: Root mean square
ICCs: Intraclass correlation coefficients
